# The Role of Choice in Weight Loss Strategies: A Systematic Review and Meta-Analysis

**DOI:** 10.3390/nu10091136

**Published:** 2018-08-21

**Authors:** Jill M. Leavy, Peter M. Clifton, Jennifer B. Keogh

**Affiliations:** 1 Dublin Institute of Technology, Dublin 6, Ireland; leajy008@mymail.unisa.edu.au; 2 Trinity College Dublin, Dublin 6, Ireland; 3 School of Pharmacy and Medical Sciences, University of South Australia, Adelaide 5000, Australia; peter.clifton@unisa.edu.au

**Keywords:** choice, weight loss, preference, diet, overweight, obesity, weight reduction

## Abstract

Effective strategies to achieve weight loss and long-term weight loss maintenance have proved to be elusive. This systematic review and meta-analysis aims to explore whether the choice of weight loss strategy is associated with greater weight loss. An electronic search was conducted using the MEDLINE (Medical Literature Analysis and Retrieval System Online, or MEDLARS Online), EMBASE (Excerpta Medica database), CINAHL (Cumulative Index to Nursing and Allied Health Literature), and PsycINFO (Database of Abstracts of Literature in the Field of Psychology, produced by the American Psychological Association and distributed on the association’s APA PsycNET) databases for clinical trials and randomized controlled trials, investigating the role of choice in weight loss strategies. A total of nine studies were identified as meeting the pre-specified criteria. All of the studies included a ‘Choice’ or preference arm and a ‘No Choice’ arm or group who did not receive their preference as a control. A total of 1804 subjects were enrolled in these studies, with weight loss observed in both experimental and control groups of all studies, irrespective of dietary intervention, study duration, or follow-up length. Twelve interventions in nine trials were used for the meta-analysis, with results indicating a greater weight loss in the control groups, 1.09 ± 0.28 (overall mean difference in weight loss between groups ± standard error; *p* = 0). There was no significant effect of duration or attrition. In this meta-analysis, the choice of weight loss strategy did not confer a weight loss benefit.

## 1. Introduction

As the worldwide prevalence of overweight and obesity increases with 39% of the worlds’ adult population overweight (BMI 25–29.9kg/m^2^) and 13% obese (BMI ≥ 30kg/m^2^) effective strategies are needed to help individuals manage their weight [1]. A systematic review and meta-analysis [2] of dietary strategies showed that all of the diets examined were superior to no diet at six months and one year. This review is consistent with evidence that calorie-reduced diets result in significant weight loss irrespective of macronutrient composition if the diet is maintained [3,4]. In order to sustain weight loss continued adherence is essential, with interventions lasting 48 months showing 3% to 6% weight loss maintenance [5]. 

Better compliance, irrespective of the type of dietary intervention employed, has been cited as the biggest predictor of weight loss [5,6,7]. With time however, compliance and motivation decreases [8]. As early attrition and weight loss are related, individuals at risk for early attrition need alternative methods to improve their retention and ultimately their weight loss success [9]. To date, decreased attrition in weight loss studies has been associated with the provision of social support, programs with supervised attendance, and programs focusing on dietary modification, as opposed to solely focusing on exercise [10]. Monetary contingency contracting in weight loss studies increases retention, which is more effective when the refund is contingent on attendance rather than actual weight loss [11]. In terms of financial rewards and retention in weight loss trials, even modest financial rewards that are associated with weight loss have successfully reduced attrition rates [12]. 

Offering individuals a choice of treatments when more than one effective treatment exists, intuitively sounds promising for improving dietary compliance. This approach has been speculated upon [2,3,4] and it has been, in part, rationalized by the behavioural choice theory [13]. Promoting participation and perceived control through offering a treatment choice has been shown to yield enhanced outcomes in medical conditions, including to mental health illnesses, colorectal cancer, breast cancer, and cardiovascular disease [14]. Allowing for change from one dietary strategy to another, thereby avoiding boredom and non-compliance, might enhance weight loss outcomes. The mechanism by which allowing a choice leads to increased success may be related to the self-efficacy and control associated with choice. 

Self-efficacy, as defined by Bandura, is a person’s perceived ability to produce a behavior [15]. Previous research has shown that increased personal efficacy is significantly related to weight loss [16] and weight loss maintenance [17]. Self-efficacy has been useful in predicting success level in behavioural weight control programs [18] and has been linked to decreased attrition [16]. Self-efficacy can be enhanced through mastery experiences and failure undermines it [15]. The availability of several successful weight loss strategies permits individuals to compare options and to choose a treatment that may be more likely to elicit a desired outcome. Making a choice gives individuals a sense of control [19]. Drawing on the theory of planned behaviour, control has proven to be the best single predictor of weight loss [20]. Therefore, allowing individuals to choose a weight loss strategy as opposed to assigning a strategy provides a sense of control, potentially enhancing weight loss outcomes. While weight cycling as a result of attempted and failed weight loss programs leads to decreased perceived control, providing a choice of alternative weight loss modalities may increase successful weight loss outcomes [21]. 

These factors may support a preferred versus a randomized treatment [22]. As these factors have shown a positive correlation with weight loss, it can be speculated that providing a choice of weight loss strategy will produce positive results. The purpose of this paper is to review studies examining the role that choice of weight loss strategy plays in the success of the intervention and to do a meta-analysis of these studies, as well as to determine any gaps in the literature that may assist with future research.

## 2. Materials and Methods

### 2.1. Data Sources

A systematic search was performed in MEDLINE, EMBASE, CINAHL, and PsycINFO for clinical trials and randomised controlled trials, investigating the role of choice in weight loss interventions, published from earliest to January 2018. Reference lists of obtained articles were also searched for relevant publications. Studies examining the role of choice in weight loss interventions were included, irrespective of the dietary strategies that were examined. Studies were limited to studies in adult humans, written in English. Key search terms were treatment prefer* and weight loss.tw OR choice and diet* prefer*.tw OR choice and weight loss.tw OR treatment prefer* and obesity.tw OR diet* prefer* and weight loss.tw OR diet* prefer* and weight management.tw OR diet* prefer* and weight reduc*. tw. The * allows for variations in the word and tw. refers to a text word, as opposed to a standard search term listed at the front of the paper.

### 2.2. Study Selection

**Figure 1:**
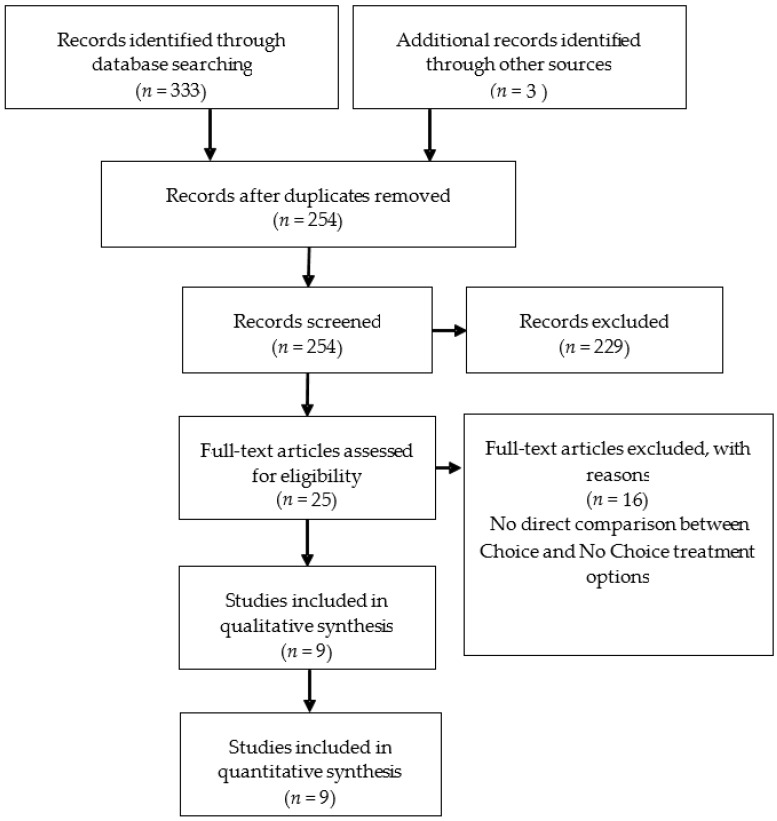
PRISMA (Preferred Reporting Items for Systematic Reviews and Meta-Analyses) flow diagram of study selection.

Studies were required to meet the following inclusion criteria: (1) original article; (2) intervention studies in humans investigating the role of choice of weight loss strategy in determining outcome; and, (3) weight loss as one of the endpoints. After the removal of duplicates, searches identified 254 articles. Of these, 229 were excluded based on title if it did not include choice or preference or imply that this was part of the design, leaving 25 possible articles; upon further examination, 16 further studies were excluded, as there was no direct comparison between a ‘Choice’ and a ‘No Choice’ treatment option. This left nine articles. Figure 1 represents a PRISMA (Preferred Reporting Items for Systematic Reviews and Meta-Analyses) flow diagram of study selection. Bias was assessed using the Cochrane Collaboration tool for risk of bias.

### 2.3. Data Analysis

A fixed and random effects meta-analysis was performed to provide an overall estimate of the difference in mean weight loss between the Choice and No-Choice interventions. Data was extracted straight in to the word table by two independent researchers. There were no disagreements and no additional data was required from the authors. Data was analysed using Comprehensive Meta-Analysis (version 2, Biostat, Inglewood, NJ, USA). For each study, the mean weight loss for each group in kg (i.e., Choice and No-Choice) was used to calculate the combined overall difference in means. Assessment of heterogeneity was made with Cochranes Q and I^2^ values and study publication bias with a funnel plot with visual inspection, Eggers regression intercept, and Duval and Tweedie’s Trim and Fill (Cochrane Q values: missing to the left in this analysis). Meta regression was performed using study duration, gender and attrition rate as predictors of the weight difference between groups.

## 3. Results

### 3.1. Systematic Review

Outlines of the nine studies, including population demographics, number of participants, participant attrition, study design, and the strategies employed, including type of intervention and weight loss outcomes, are presented in Figure 1.

Of the studies included, six used two dietary strategies—low-carbohydrate vs. low-fat [23,24]; group therapy vs. individual therapy [25]; vegetarian vs. standard omnivorous diet [26]; self-control vs. determination raising [27]; and, group vs. telephone contact [28]. Two used three dietary strategies—Commonwealth Scientific and Industrial Research Organisation (CSIRO) vs. South Beach vs. Mediterranean diet [29] and nutrition education vs. behaviour management vs. exercise [30]. One study employed six dietary options—three commercial and three traditional weight loss strategies [31], with either allocation or free choice of these diets. A total of 1804 subjects enrolled in these studies collectively and were randomised, with overall attrition ranging from 11% to 42.7% at study end. See Table 1 (‘*n*’ columns) for study completers.

All of the studies in this review were deemed to have a similar risk of bias (at a study level), as assessed by the Cochrane Collaboration’s tool for assessing risk of bias. No two studies were identical in duration. Interventions ranged from ten weeks to two years. Of the nine studies included, two had only female participants [27,28], whilst five had predominantly female participants [23,25,26,30,31]. One study had a majority of male participants [24] and one had an even distribution of males and females [29]. The age of study participants was similar in six studies [23,25,26,28,30,31], while two studies recruited older participants [24,29] and one study had younger participants [27]. One sample population was entirely veterans [24]. Two studies offered their ‘Choice’ participants the option to change dietary strategy [24,29] at an intermediate point in the intervention with five participants in both cohorts opting to change. Eight [24,25,26,27,28,29,30,31] of the nine studies dichotomised results, making direct comparisons between the ‘Choice’ or preference and ‘No Choice’ or no preference arms. Borradaile [23] trichotomised results, stratifying the participants into ‘Treatment congruent’ (those who were allocated to their preferred diet), ‘Treatment incongruent’ (those who were not allocated to their preferred diet), and those who expressed ‘No strong preference’.

Prior to and independently of randomisation into dietary strategy arms, five studies assessed participant treatment preference [23,25,26,27,28]. The remaining studies randomised participants into either a ‘Choice’ or ‘No Choice’ arm. One study [24] assessed food preference while using the Geiselman Food Preference Questionnaire at the screening visit. These data were then summarised for participants in the ‘Choice’ group and used to indicate with which of the two diet options individual preferences aligned. One study provided participants with vouchers [31] and monetary contingency contracting was used in three studies [27,28,30].

Of the nine studies included, there was no statistically significant difference in weight loss between the ‘Choice’ and ‘No Choice’ groups in six studies [24,25,27,28,29,31]. Three studies [23,26,30] showed a statistically significant difference in mean weight loss between groups, with the ‘No Choice’ groups losing significantly more weight than the ‘Choice’ groups. Whilst there was weight loss in both arms at the endpoint, weight loss varied between studies. This may be partially explained by study duration and sample size (*n* = 12–740), limiting the opportunity to make direct comparisons. Other possible explanations include differing attrition rates, population demographics, and the dietary strategies used. Only two studies measured dietary composition and two studies measured physical activity and there was no difference between interventions for either variable.

### 3.2. Meta-Analysis

**Figure 2:**
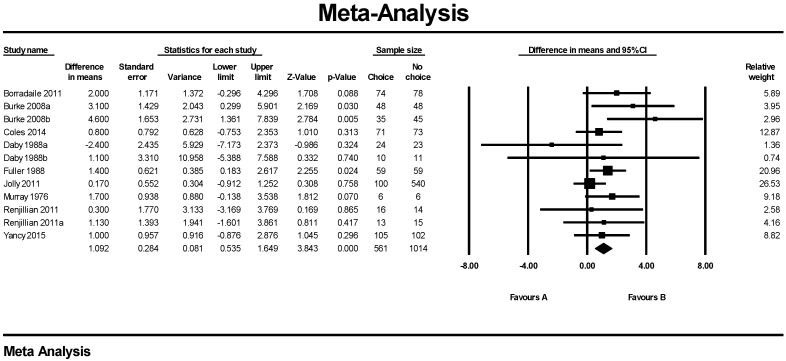
Forest Plot from fixed meta-analysis for weight in Kg. A = Choice, B = No-Choice.

**Figure 3:**
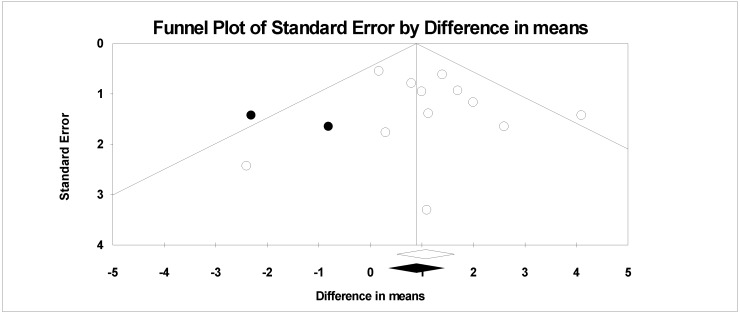
Funnel plot observed and imputed values from Duval and Tweedie’s trim and fill.

Twelve interventions in nine trials were used for the meta-analysis, with results indicating a greater weight loss in the ‘No Choice’ control groups, fixed effects 1.09 ± 0.28 kg (overall mean difference in weight loss between groups ± standard error; *p* = 0.000) (Figure 2). Random effects analysis was very similar (1.17 ± 0.33 kg *p* = 0.000). There was no significant heterogeneity with a Q value of 12.9 *p* = 0.297 and an I^2^ of 15.0. Removal of one study at a time had no effect on the mean value. Cumulative analysis achieved the final mean change after the addition of 7 studies with no change with the next five studies. Funnel plot (Figure 3) shows no bias in published studies with or without trimmed studies (Q value 12.9 without and 20.8 with right side trimmed values). Egger’s regression had a 2-tailed *p* value of 0.4. There was no significant effect of duration, gender, or attrition. Meta-analysis of the data clearly favors no-choice.

## 4. Discussion

The results of this systematic review of the available literature provide evidence that any weight loss strategy leads to a weight reduction. However, contrary to conceptual predictions, weight loss is not greater when participants were given a choice, as opposed to being assigned a dietary strategy. 

Two main study designs were implemented across studies; randomization of participants into ‘Choice’ and ‘No Choice’ arms, only assessing participant preference in the ‘Choice’ groups, and ranking participant treatment preference independently but prior to randomization into dietary groups. Five studies ranked preference using Likert scales. Two of these studies excluded participants with a slight or no preference for one diet over another [25,26]. One study advised those with a strong aversion to one diet, not to enroll [24]. Another study [23], which was based on qualitative feedback decided to use three categories to describe preference, treatment congruent, treatment incongruent, and no strong preference, as above descried. Interestingly, in this study, it was the group that did not have a strong preference that lost the most weight. Borradaile hypothesizes that this was due to an internalized responsibility for weight loss in this group. This was supported by qualitative data collected (i.e., ‘No preference because I was willing to give either diet 100%’). Therefore, perhaps had this cohort been included in Renjilian and Burke’s studies, a different outcome may have been observed. The exclusion of those without a strong preference from other studies limits the ability to directly compare these studies.

In Yancy’s study [24], although participants were randomized to ‘Choice’ or ‘No Choice’ groups, food preferences of the participants were assessed. Individuals were advised to choose and follow a diet consistent with their food preference. Nonetheless, this did not produce a result that favoured the ‘Choice’ group, likely related to the behavioural choice theory. Participants’ preferred foods are presumably those that they find most palatable [32]. Several studies have shown a positive relationship between palatability and food intake [33,34,35]. Whilst preference was facilitated in this trial to enhance participant retention, the behavioural choice theory states that choice will only be successful in modifying a deficit behaviour (i.e., a behaviour not yet learned). Providing a choice to participants which is informed by their food preference may therefore not be effective, illustrated by the results of this study where giving a choice did not elicit a more favorable outcome. This is supported by qualitative data from Borradaile’s study [23], which suggests that participants chose their diet based on their preferred foods, which may have led to less weight loss in this ‘Choice’ group. 

The studies that randomized participants to ‘Choice’ and ‘No Choice’ groups encountered challenges in study processes. Murray randomized participants to a ‘Choice’ and ‘No Choice’ group, which was a choice of time, rather than treatment [27]. Initially, 27 applicants were selected, but due to scheduling conflicts only 12 participants commenced the trial. Applicants were asked to select a preferred treatment type and preferred treatment time. As researchers could only match six pairs based on preferred time and baseline weight, only 12 out of an eligible 27 individuals participated. In Burke’s trial a 3:2 ratio preference-yes: preference-no was selected. The preference-yes group was over sampled as a smaller cohort was expected to choose the lacto-ovo vegetarian diet, based on results of a pilot study. Following randomization, a larger standard diet group resulted, which lead to the disqualification of 15 participants from the preference-yes, standard diet group. The alteration in study design of these two studies, highlights the need to interpret the results with caution. 

Two studies gave the option to alternate diets [24,29]. Coles’ [29] participants, could alternate at any time, with permission from the researcher. Yancy [24] permitted this change only at week 12. In each of these studies, five participants opted to change from their original diet. Coles reported this change between 8 and 20 weeks—4 female and 1 male. There is no specific indication from either study as to the success of participants who alternated dietary strategies. Despite the availability of this choice, results from neither study demonstrated a significant difference in mean weight loss between groups. There was no significant difference in attrition rates between groups in one study offering this choice, and rate of attrition in the two groups was not explored in the second study.

Attrition ranged from 11% to 42.7% across all studies, independent of study duration. This is consistent with previous reports of a 10% to 80% attrition from weight loss studies [36,37,38,39]. Jolly [31] provided all of the participants with twelve weeks of vouchers for their assigned or chosen weight loss intervention. Interestingly, the attrition rate in this study was the least (11.1%). However, this 2011 study was short and that may have contributed to its low attrition rate. A positive association has been speculated between treatment time and weight loss [9,40,41], however a meta-analysis showed no relationship between treatment time or length of follow-up and retention rate [42]. Three trials [27,28,30] included a participation fee. This money was either returned according to attendance, not contingent upon weight loss, or used as raffle prize. Previous research has identified a positive effect of contingency contracting to decrease attrition in weight loss studies, when compared with free or paid participation, which is not supported in this review [43]. Fuller [30] and Coles [29] provide reasoning behind participant cessation in their trials, the most common being ‘scheduling conflicts’, with the second most common reason being due to participants not achieving their goals. Daby [28] explores the reasons behind some of the attrition, however this study does not account for all drop-outs. 

When comparing attrition in ‘Choice’ vs. ‘No Choice’ groups, Fuller [30] and Daby [28] both report a statistical difference in drop-out rates between groups. Both report higher attrition in their ‘No Choice’ groups. This is congruent with previous research, which has concluded that participants who receive their preferred treatment are half as likely to drop out of the intervention when compared to their counterparts who do not receive their preferred treatment [44]. This discrepancy may introduce bias, favouring assigned subjects in Fuller and Daby’s studies. Some ‘Choice’ participants may only have remained due to commitment to treatment choice as opposed to commitment to weight loss. Remaining subjects in the ‘No Choice’ group may have been more committed to the final outcome, irrespective of the dietary regimen that is required to achieve their weight loss targets and may therefore have been more resilient, resulting in a more favorable weight loss outcome in this population. However, had this been the case it would be expected that the variance in weight loss would be smaller in the ‘No Choice’ cohort, which was not the case in either study. Nonetheless, when Daby [28] explored the relationship between attrition and weight loss, there was significantly less weight loss in the ‘no shows’ versus completers at program end, using last weight carried forward. However, at follow-up, there was no difference in weight loss between completers and non-completers. Although decreasing attrition rates is important, this highlights the need to promote weight maintenance in the long term beyond program end. 

A recurrent theme in seven [23,25,26,27,28,30,31] of the nine selected studies is the underrepresentation of males, with male participation overall ranging from 0% to 63% of the total study populations. This gender imbalance could limit the generalizability of these findings, as previous research has shown that men lose significantly more weight than women in weight loss trials [45,46]. Coles [29] noted that weight loss in men exceeded that of the women in the ‘No Choice’ group, and vice versa in the ‘Choice’ group where females excelled (*p* = 0.002). Jolly [31] reported gender difference in choice of diet, with 81% of females choosing a commercial program versus 47% of males opting for this choice. This is congruent with findings that commercial programs are perceived as more female oriented [47]. Coles [29], likewise, reported gender differences in dietary preference, men most frequently opted for the South Beach diet and women showed a strong preference for the CSIRO diet. While current global statistics suggest that overweight and obesity in men is increasing [1], overallan average male participation in weight loss trials has been estimated at 27% [48]. With male obesity rising more rapidly than female figures in high-income countries [49], the underrepresentation of males in weight loss studies is of concern. Gender specific interaction with choice in weight loss strategies is a largely unexplored area and it could yield interesting results given the enduring weight challenges in both genders.

Initial weight of participants in each study varied both within and between trials. In the included studies, all but one reported no difference in baseline weight characteristics of the ‘Choice’ and ‘No Choice’ groups. Burke [26] reported a significantly higher baseline weight in participants in the group that did not receive their preference (*p* = 0.01). This may be explained by stratification for gender, ethnicity and diet preference prior to randomization and failure to account for baseline weight. This study showed a significantly greater weight loss in the no-preference cohort. Previous research regarding the effect of baseline weight on weight loss is inconclusive. Studies suggest that the magnitude of weight loss is not related to baseline body weight [50] or BMI [51], yet preoperative BMI has shown to be inversely related to weight loss post weight loss surgery [52,53]. Therefore, the significant difference in weight loss observed in this study is unlikely to be due to baseline weight, nor to other demographic characteristics, as these were reportedly consistent between the two groups. When comparing trials baseline weights differed, however this did not influence the magnitude of weight lost.

Potential bias may arise from the analysis of participant data who have remained and not dropped out because of failure to succeed. Assuming, however, that the inclination to enroll subjects likely to be successful is distributed evenly across all intervention groups, trial comparisons are still appropriate. Additionally, heterogeneity in dietary strategies, overall study design, and sample sizes limits the applicability of the findings, and this is shown by the small number of studies that met the inclusion criteria. Despite these limitations, this review provides a valuable insight into the role that the choice of dietary intervention plays in weight loss. 

Several factors may have influenced the findings of this review including the inclusion criteria and potential publication bias. However, limitations are predominantly reflected by the limitations in the primary studies, such as study design, methodology, and the assumption that the evaluation techniques are consistent across studies. All of the studies in this review were deemed to have a similar risk of bias (at a study level), as assessed by the Cochrane Collaboration’s tool for assessing risk of bias.

## 5. Conclusions

This review shows that, at present, there is no evidence to support the notion that giving overweight and obese individuals a choice of treatment leads to superior weight loss than prescribing a specific dietary regimen. Furthermore, attrition rates were similar in ‘Choice’ and ‘No Choice’ arms of the included studies, suggesting no advantage or disadvantage to providing a choice in terms of participant retention and adherence.

## 6. Limitations

Study duration and the types of dietary strategy used varied significantly, limiting the ability to make direct comparisons between studies. This area requires further research, including studies of similar duration, population demographics, and comparable dietary strategies to fully appreciate any significant benefit that providing a dietary choice may have on weight loss outcomes.

## Figures and Tables

**Table 1:** Study outlines.

Author (Year)	Study Design	Study Population	Start (*n*)	End (*n*) (% of Attrition)	Strategy	Option to Change Diet	Study Outcome * Mean ± Standard Deviation ** Mean (95% Confidence Interval)
Borradaile et al. [23]	RCT, over 2 years. Baseline data collected, weekly data for 20 weeks, fortnightly for week 20–40 and every other month for remainder of 2 years.	18–65 years BMI 30–40 kg/m^2^	250	157 (37.2%)	Treatment preference assessed prior to, but independent of, randomization into either low-carbohydrate or low-fat diet. Preference congruent (*n* = 74); no strong preference (*n* = 98); preference incongruent (*n* = 78).	No	At 24 months ** Treatment congruent (a); ↓7.7 kg (↓9.3 to ↓6.1) Treatment incongruent (b); ↓9.7 kg (↓11.4 to ↓8.1) No strong preference (c); ↓11.2 kg (↓12.6 to ↓9.7) *p* = 0.04 (a) and (b) *p* = 0.0004 (a) and (c)
Burke et al. [26]	RCT. Initial weekly sessions for 6 months, every 2 weeks for months 7 to 9, and monthly for months 10–12. Baseline data collected and every 6 months for 18 months.	18–55 years BMI 27–43 kg/m^2^	176	132 (25%)	Participants ranked their preference for a Lacto-ovo-vegetarian diet (LOV-D) or a Standard calorie- and fat-restricted diet (STD-D) on a 3-point Likert scale. Those with mild or no preference were disqualified. Participants were randomized into four groups, preference-yes STD-D (*n* = 48); preference-no STD-D (*n* = 48); preference-yes LOV-D (*n* = 35); preference-no LOV-D (*n* = 45).	No	At 18 months * Preference-yes STD-D; ↓4.8 kg ± 6.1 Preference-yes LOV-D; ↓4.8 kg ± 6.2 Preference-no STD-D; ↓7.9 kg ± 7.8 Preference-no LOV-D; ↓9.4 kg ± 8.1 *p* = 0.02 difference between preference-yes and preference-no
Coles et al. [29]	RCT, over 12 months. Baseline data collected, data obtained every 2 weeks until month 3, then every 6 weeks until 12 months, and 3, 6 and 12 month visits.	40–75 years BMI > 27 kg/m^2^	144	96 (33.3%)	Participants randomized to ‘Choice’ or ‘No Choice’ groups. ‘No Choice’ group (*n* = 73) was placed on a set weight loss diet (CSIRO), no change permitted. ‘Choice’ group (*n* = 71) chose from the CSIRO (*n* = 34); South Beach (*n* = 11) or Mediterranean diet (*n* = 26).	Yes at any time	At 12 months * ‘No Choice’ group; ↓3.5 kg ± 4.5 kg ‘Choice’ group; ↓2.7 kg ± 5 kg *p* > 0.05
Daby [28] ^1^	RCT. Phase I; weekly group meeting for 5 weeks. Phase II; biweekly meetings for 8 weeks and then monthly for 2 months OR weekly phone call. Baseline weight, weight at group meetings, at end of phase 2 and at trial end.	20–60 years 40%–150% above ideal body weight Females	82	47 (42.7%)	All participants attended Phase I. Participants then expressed preference for ‘Group’ based or ‘Telephone’ based interventions. Participants were randomized into two groups independent of preference. Four groups resulted, group-yes (*n* = 24); group-no (*n* = 23); telephone-yes (*n* = 10); telephone-no (*n* = 11).	No	At 33 weeks * Group-yes; ↓15.1 lbs ± 19.3 (6.8 kg ± 9) Group-no; ↓9.7 lbs ± 16.7 (4.4 kg ± 7.6) Telephone-yes; ↓7.6 lbs ± 11.8 (3.4 kg ± 5.4) Telephone-no; ↓9.86 ± 20.2 (4.5 kg ± 9.1) *p* > 0.05
Fuller [30] ^2^	RCT over 10 weeks. Baseline weight was obtained and weekly weights thereafter.	24–65 years Overweight	118	73 (38.1%)	Participants randomized to ‘Choice’ or ‘No Choice’ groups. ‘Choice’ group chose between nutrition education (*n* = 11), behaviour management (*n* = 22) or exercise (*n* = 19) for weight loss. ‘No Choice’ were randomly assigned to nutrition education (*n* = 15); behaviour management (*n* = 15) or exercise (*n* = 13) for weight loss.	No	At 10 weeks ‘Choice’ group; ↓4.5 lbs (2 kg) ‘No Choice’ group; ↓7.4 lbs (3.4 kg) *p* = 0.026
Jolly et al. [31]	RCT over 12 weeks. Baseline data was collected during week 1 and again at 3 months.	≥18 years BMI ≥ 30 kg/m^2^ with no obesity related comorbidities or ≥25 kg/m^2^ if South Asian BMI ≥ 28 kg/m^2^ with obesity related comorbidities or ≥23 kg/m^2^ if South Asian.	740	658 (11.1%)	Participants were randomized to; Weight Watchers (*n* = 100); Slimming World (*n* = 100); Rosemary Conley (*n* = 100); group based, dietetics led program (*n* = 100); general practice one to one counselling (*n* = 70); pharmacy led one on one counselling (*n* = 70); choice of any of the six programs (*n* = 100) or a comparator group provided with 12 vouchers for a local fitness center.	No	At 3 months ** WW ↓5.2 kg (↓4.2 to ↓6.1) SW ↓4.3 kg (↓3.3 to ↓ 5.2) RC ↓5.3 kg (↓4.2 to ↓6.4) SD ↓3.2 kg (↓2.3 to ↓4.1) GP ↓2.2 kg (↓0.7 to ↓3.7) P ↓2.8 kg (↓1.4 to ↓4.2) Choice ↓3.8 kg (↓2.9 to ↓4.7) Control ↓3 kg (↓1.8 to ↓4.1) *p* > 0.05 difference between ‘Choice’ and ‘No Choice’ groups
Murray et al. [27]	RCT, over 6 months. Baseline data and weekly data was collected for 9 weeks followed by a 3 month and 6 month data collection.	≥21 years >165 lbs (74.8 kg) Females	12	9 (25%)	Participants indicated their treatment preference. Patients were independently assigned to follow the self-control method (*n* = 6) or determination raising (*n* = 6) method by chance.	No	At 6 month follow-up Preference-yes group; ↓2.8 lbs (1.3 kg) Preference-no group; ↓6.5 lbs (3 kg) *p* > 0.05
Renjilian et al. [25]	RCT trial over 26 weeks. Baseline data was obtained, and the weekly data collected for 26 weeks.	21–59 years BMI 28–45 kg/m^2^	75	58 (22.7%)	Participants indicated preference for Individual vs. Group therapy using a 6-point Likert scale, those who had a ‘slight’ preference were excluded from the study. Participants were randomized to one of the two groups. Four groups resulted; Preference-yes group (*n* = 16); preference-yes individual (*n* = 13); preference-no group (*n* = 14); preference-no individual (*n* = 15).	No	At 26 weeks * Preference-yes group ↓10.9 kg ± 4.06 Preference-no group ↓11.2 kg ± 5.6 Preference-yes individual ↓8.48 kg ± 3 Preference-no individual ↓9.61 kg ± 4.17 *p* > 0.05
Yancy et al. [24]	Doubly randomised, preference trial over 48 weeks. Baseline data was obtained, every 2 weeks for 24 weeks, then every 4 weeks for 24 weeks.	<75 years BMI ≥ 30 kg/m^2^ * Participants were veterans	207	175 (15.5%)	Participants completed a food preference questionnaire. Participants were randomized to a ‘Choice’ or ‘No Choice’ arm. The ‘Choice’ group received information about their food preferences in relation to 2 diet options-low carbohydrate (*n* = 61) or low-fat (*n* = 44), before choosing one. The ‘No Choice’ group were randomly assigned to the low-carbohydrate (*n* = 53) or low–fat (*n* = 49) diet.	At week 12	At 48 weeks ** ‘Choice’ arm ↓5.7 kg (↓4.3 to ↓7) ‘No Choice’ arm ↓6.7 kg (↓5.4 to ↓8) Mean difference ↓1.1 kg (↓2.9 to ↓0.8) between groups *p* = 0.26

RCT = Randomised controlled trial, CSIRO = Commonwealth Scientific and Industrial Research Organisation, ^1^ Unpublished thesis from depository of Vanderbilt University, ^2^ Unpublished thesis from depository of Michigan State University. * mean ± standard deviation, ** mean (95% confidence interval).
